# Newer Insights Into Fetal Growth and Body Composition

**DOI:** 10.3389/fendo.2021.708767

**Published:** 2021-07-22

**Authors:** Satoru Ikenoue, Yoshifumi Kasuga, Toyohide Endo, Mamoru Tanaka, Daigo Ochiai

**Affiliations:** Department of Obstetrics and Gynecology, Keio University School of Medicine, Tokyo, Japan

**Keywords:** DOHaD, fetal ultrasound, fetal body composition, fetal subcutaneous fat, fractional limb volume, fetal growth restriction, macrosomia, predisposition

## Abstract

Based on epidemiological and experimental evidence, the origins of childhood obesity and early onset metabolic syndrome can be extended back to developmental processes during intrauterine life. It is necessary to actively investigate antecedent conditions that affect fetal growth by developing reliable measures to identify variations in fetal fat deposition and body composition. Recently, the resolution of ultrasonography has remarkably improved, which enables better tissue characterization and quantification of fetal fat accumulation. In addition, fetal fractional limb volume has been introduced as a novel measure to quantify fetal soft tissue volume, including fat mass and lean mass. Detecting extreme variations in fetal fat deposition may provide further insights into the origins of altered fetal body composition in pathophysiological conditions (i.e., fetal growth restriction or fetal macrosomia), which are predisposed to the metabolic syndrome in later life. Further studies are warranted to determine the maternal or placental factors that affect fetal fat deposition and body composition. Elucidating these factors may help develop clinical interventions for altered fetal growth and body composition, which could potentially lead to primary prevention of the future risk of metabolic dysfunction.

## Introduction

Fetal growth is an important predictor of perinatal outcomes. Previous studies have also shown that birth weight is associated with future risk of obesity and metabolic dysfunction in the offspring. Barker et al. reported that low birthweight infants are predisposed to cardiovascular disease in adulthood (1). Maternal undernutrition during pregnancy leads to increased risks of cardiovascular disease in the offspring in later life (2). Meanwhile, the infants of mothers with gestational diabetes have increased adiposity (3). Large-for-gestational age (LGA) neonates born to mothers with gestational diabetes are predisposed to early onset metabolic syndrome (4).

These experimental and epidemiological evidence have shown that the origins of early onset metabolic syndrome can be extended back to developmental processes during intrauterine life (Developmental Origins of Health and Disease) (5–7). Therefore, it is necessary to actively investigate the antecedent conditions that affect fetal growth by developing reliable measures to identify variations in fetal growth and body composition.

## Body Weight and Body Composition

Total body weight and height has been traditionally used as the gold standard for the measurement of body size and nutritional status. Recently, the concept of body composition has been introduced, which is composed of fat mass, lean mass, and bone mineral content.

### Neonatal Body Composition

Previous studies on infant body size have mainly relied on height- and weight-based measures (i.e. weight for length, body mass index, or ponderal index). However, these parameters are indirect measures of fat mass or lean mass, and only moderately correlate with percent body fat (8). Newborns with decreased percentage body fat are reported to be at risk of hypoglycemia and short-term morbidity (9). Small-for-gestational age (SGA) newborns, who are supposed to be predisposed to thrifty phenotype, have lower body fat percentage compared to appropriate-for-gestational age infants (10). Higher neonatal body fat percentage is also associated with increased adiposity in childhood (11). These reports suggest that neonatal body composition and body fat percentage could be a more specific marker of future risk of metabolic syndrome as compared with birth weight (11, 12).

The body fat percentage of neonates is higher in humans than in other mammals. It is important for human neonates to prioritize adipose tissue accumulation because adipose tissue is an important buffer against limited nutrient supply soon after birth, and could be utilized as one of the brain’s energy resources (13). Indeed, the infant brain requires approximately half of the total energy needs, and ketone bodies derived from adipose tissue can provide a quarter of this requirement (14). Consequently, although neonatal fat mass constitutes only 14% of birth weight, it accounts for a larger variation (46%) in birth weight (15, 16). In contrast, the ponderal index explains only 22% of the variation in birth weight and correlates poorly with body fat percentage (16).

Dual-energy X-ray absorptiometry and air displacement plethysmography have recently been used as gold standards for measuring body composition and body fat percentage in infants (17).

### Ultrasound-Based Measures of Fetal Weight

Fetal ultrasonography is commonly used to evaluate fetal growth in clinical practice, and most studies use conventional biometry (estimated fetal weight). Estimated fetal weight has been reported to be a useful predictor of fetal macrosomia (18), or fetal growth restriction with decreased percent body fat (19). However, the estimated fetal weight can fluctuate by approximately 15% compared to the actual weight and has poor accuracy, especially for fetuses with growth restriction or macrosomic infants (20). A possible explanation is that the estimated fetal weight is an indirect measure of fat mass or lean mass, and has only a modest association with newborn adiposity (21). However, few studies have incorporated fetal fat mass or lean mass measures into formulas that estimate fetal weight. There have recently been remarkable improvements in the resolution of fetal ultrasonography, which enables better tissue characterization and quantification of fetal fat mass and lean mass. Several studies have been conducted to assess fetal body composition (22) and its correlation with newborn adiposity (**Table 1**).

**Table 1:** Fetal fat mass measures predicting newborn adiposity.

Author, publication year	N	Newborn adiposity measures		Gestational age at fetal ultrasonography	Fetal biometry	Correlation coefficient	p value	Covariates
		Parameter	Device					
Ikenoue et al. (23)	109	%BF	DXA	20, 30 weeks	Arm percent fat area (30 weeks)	0.45	p<0.001	GA, Parity, BMI, GWG, SES, Ethnicity, Obstetrical complications
					Thigh percent fat area (30 weeks)	0.26	p<0.05	
					FAST (30 weeks)	0.21	p<0.05	
O’Connor et al. (24)	62	Fat mass	ADP	28, 33, 38 weeks	FAST (38 weeks)	−	p<0.001	smoking
					Thigh fat thickness (38weeks)	−	p=0.004	
					Thigh fat thickness (28weeks)	−	p=0.023	
Buhling et al. (25)	172	Skinfold thickness	Anthropometry*	37− weeks	FAST	0.58	p<0.001	BMI, placental site, amniotic fluid volume
					Thigh fat thickness	0.64	p<0.001	
Knight et al. (26)	106	%BF	ADP	36−40 weeks	Arm fat area	−	p<0.001	−
Lee et al. (27)	87	%BF	ADP	38 weeks (mean)	Fractional thigh volume	0.68	p<0.001	Age, parity, GA, sex, ethnicity, Obstetrical complications
					Fractional arm volume	0.62	−	
					Estimated fetal weight	0.55	−	
					Abdominal circumference	0.50	−	
Moyer-Mileur et al. (21)	47	%BF	ADP	35 week	Estimated fetal weight	0.33	p<0.05	Parity, BMI, GWG, SES
					Abdominal circumference	0.37	p<0.05	
Bernstein et al. (28)	36	%BF	Anthropometry*	19−40 weeks	Thigh fat area	0.63	p<0.001	Age, Parity, BMI, GA, GWG, sex
					Arm fat area	0.45	p<0.05	

%BF, percent body fat; DXA, Dual-energy X-ray absorptiometry; ADP, air displacement plethysmography; FAST, fetal abdominal subcutaneous tissue; GA, gestational age; BMI, body mass index; GWG, gestational weight gain, SES, socioeconomical status.

*Sum of subcutaneous skinfold thickness.

### Ultrasound-Based Measures of Fetal Body Composition

Histologically, fetal fat is observed in early gestation (29). However, measures of fetal fat mass or lean mass by ultrasonography are mostly obtained after 20 weeks of gestation because enlargement and accumulation of adipocytes accelerate in the second half of pregnancy (30). Since 70-90% of total body fat in human infants is subcutaneous and not visceral (31), subcutaneous fat mass is measured for evaluating fetal adiposity. Fetal fat mass can be reliably and reproducibly measured using ultrasonography at the mid-upper arm, mid-thigh, and abdomen (32).

#### Fetal Fat Mass Measures in the Upper-Arm and Thigh

In 1985, Jeanty et al. reported the measurement of subcutaneous fat thickness in the arm and thigh (33). This method yields substantial measurement error because the subcutaneous fat thickness in the limb is not continuous around the limb. In 1997, Bernstein et al. measured the fat area on the arm and thigh, instead of the fat thickness at term gestation. The fat area on the arm and thigh were quantified by subtracting the lean mass area from the total cross-sectional area at the midpoint of the humerus or femur. They reported a significant correlation between the fat area of the limb and newborn fat mass (28).

#### Fetal Fat Mass Measures in the Abdomen

In previous studies, abdominal subcutaneous fat thickness was measured as the hyperechoic region anterior to the margins of the ribs. The widest point was selected in the anterior third of the abdominal circumference (34). In 1997, Petrikovsky et al. reported that anterior abdominal wall thickness is useful for ruling out fetal macrosomia (35). Subsequently, in 1999, Gardeil et al. measured anterior abdominal wall thickness as a predictor of fetal growth restriction (34). An increasing amount of evidence has been gathered over the last 20 years in the assessment of fetal body composition and fat mass (22).

#### Fetal Fat Mass Measures in Other Parts

In addition to the three major sites (upper-arm, thigh and abdomen), fetal fat is deposited in other areas such as the cheek, ribs, and buttocks (22, 36, 37). Abramowicz et al. has reported that fetal cheek-to-cheek diameter is useful in the prediction of birth weight and mode of delivery (36, 38, 39). Matsumoto et al. reported the fetal nutrition score, derived from fetal subcutaneous tissue present at face, ribs and buttocks. The fetal nutrition score significantly correlated with neonatal nutrition score derived from face, ribs, thigh and buttocks (37). However, the accuracy of quantitative measurement of fat mass in these areas has not been validated.

#### Fetal Fractional Limb Volume

3D ultrasonography refers specifically to the volume rendering of ultrasound data, and is now widely used in the clinical practice including prenatal diagnosis. Fetal fractional limb volume measured by three-dimensional ultrasonography was introduced by Lee et al., defined as the cylindrical limb subvolume based on the central 50% of the total humeral or femoral length (40). It is a reproducible measure for quantifying fetal soft tissue volume, including fat mass and lean mass (41, 42). Usually, before starting fractional limb volume measures, sonographers are required to measure 20-30 training data sets. The learning curve for technically satisfactory measurements of fractional limb volume is quickly achieved without difficulty (43). Recently, automated fractional limb volume measures has also been investigated (44). Fractional limb volume is useful to improve birth weight estimation (43) and accounts for a greater proportion of variation in neonatal body fat percentage than conventional fetal measures such as estimated fetal weight (27). The growth trajectory of fetal soft tissue volume (especially fat mass) accelerates early in the third trimester (45, 46), which coincides with the accelerated growth of fractional limb volume around 30 weeks of gestation (44, 47, 48). We recently reported greater fetal fractional arm volume among mothers with gestational diabetes in late gestation (49).

### Maternal-Fetal Factors That Affect Fetal Body Composition

Maternal pregravid body mass index (BMI) and diabetes are well known determinants of fetal growth, in particular fetal adiposity (50, 51). Infants born to women with higher BMI have increased percentage body fat as compared to infants born to women with normal BMI (52, 53). Overweight or obese women with normal glucose tolerance levels still have infants with increased adiposity (50). Maternal gestational weight gain and presence of gestational diabetes have been associated with increased fetal abdominal subcutaneous fat thickness (54). Moreover, several biomarkers (maternal systemic interleukin-6, cord blood leptin and insulin-like growth factor) potentially affect fetal body composition (55, 56).

### Fetal Liver Blood Flow Volume and Infant Body Composition

Fetal liver blood flow volume has recently emerged as one of the determinants of fetal growth and subsequent infant body composition (57, 58). The fetal liver is the primary site where nutrient interconversion and *de novo* synthesis occur (59). Hence, variation in the relative distribution of umbilical venous blood flow shunting either through the ductus venosus or through the fetal liver has been proposed as a mechanism of fetal adaptation to intrauterine conditions (23, 60, 61).

Several studies have been conducted investigating the association between fetal liver blood flow volume and maternal pregravid BMI (62), gestational weight gain (63), serum glucose level (64–66), and fetal growth restriction (67). More recent report showed the correlation between fetal liver blood flow volume and placental corticotrophin releasing hormone, which is a paracrine determinant of the placental vasculature (68). Considering these previous reports, assessing fetal liver blood flow volume may help better understand the mechanisms influencing fetal growth and body composition.

## Future Perspectives

Further studies are warranted to determine the association between ultrasound-based measures of fetal body composition and metabolic dysfunction in later life. It is also important to investigate the factors (maternal demographic background, metabolic status including dyslipidemia and dysglycemia, and placental transporters of the nutrients) that affect fetal body composition and fat deposition (69).

Fetal fat mass and fractional limb volume could also be surrogate markers of fetal nutritional status, to distinguish constitutionally small/large fetus from malnourished/overnourished fetus. Physiological diversity and heterogeneity in fetal growth velocity patterns (especially in the third trimester of gestation) has been reported (70). Additionally. the growth trajectory of fetal soft tissue volume (especially fat mass) accelerates early in the third trimester (45, 46). Based on these, sequential measures of fetal fat mass and fractional limb volume in the third trimester (e.g. every 2-4 weeks) could be clinically useful to distinguish constitutionally small/large fetus from malnourished/overnourished fetus. These can help better understand the “thrifty” or “drifty” phenotype of the fetus, both of which are predisposed to the metabolic syndrome in later life. Further studies should be conducted to evaluate how these findings translate into clinical interventions for altered fetal growth and body composition. This could potentially lead to the primary prevention of future risk of metabolic dysfunction.

## Conclusion

An ultrasound-based measure of fetal fat mass has been established that provides new insights into the evaluation of fetal growth and body composition, and its relationship with newborn adiposity. The ability to detect extreme variations in fetal fat deposition may help understand alterations in fetal body composition in pathophysiological conditions, such as fetal growth restriction or fetal macrosomia. Further studies are warranted to elucidate the maternal or placental factors that affect fetal fat deposition and newborn body composition. Elucidating these factors could help develop clinical intervention strategies for altered fetal growth and body composition, which potentially lead to primary prevention of the metabolic dysfunction in later life.

## Author Contributions

SI researched data, wrote the manuscript. SI, YK, TE, MT, and DO contributed to discussion and reviewed the manuscript. All authors contributed to the article and approved the submitted version.

## Funding

This work was funded by the Japan Society for the Promotion of Science, KAKENHI Grant Number 20K18231.

## Conflict of Interest

The authors declare that the research was conducted in the absence of any commercial or financial relationships that could be construed as a potential conflict of interest.
